# Re: Stress and musculoskeletal pain in physiotherapists during the pandemic depend on a plethora of influencing factors

**DOI:** 10.1186/s12998-023-00525-w

**Published:** 2024-02-16

**Authors:** Nathan Weiss, Eva Skillgate, Iben Axén

**Affiliations:** 1 Unit of Intervention and Implementation Research for Worker Health, Institute of Environmental Medicine, Karolinska Institutet, 171 77 Stockholm, Sweden; 2 Department of Health Promotion Science, Musculoskeletal and Sports Injury Epidemiology Center, Sophiahemmet University, 114 86 Stockholm, Sweden; 3 Naprapathögskolan-Scandinavian College of Naprapathic Manual Medicine, 114 19 Stockholm, Sweden; 4 Et Liv I Bevegelse, Oslo, Norway

The authors would like to thank Finsterer and colleagues for their interest in our study [1].

We agree that if the aim was to investigate whether psychological distress and musculoskeletal pain increased during the COVID-19 pandemic, and to understand underlying causal factors, one would need to consider a long row of potential risk factors and confounders, and to have a control group, as well as prospective data.

The aim of this cross-sectional study was not to deepen the knowledge about causality nor to investigate what factors were “influencing the development of psychosocial stress and musculoskeletal pain”– but to “*describe the prevalence of psychological distress and musculoskeletal pain, and to investigate factors potentially associated with high psychological distress and activity-limiting musculoskeletal pain in clinically active chiropractors and naprapaths during the second wave of the COVID-19 pandemic in Sweden*.” With that in mind, we do not agree with the limitation of our study suggested by Dr. Finsterer. We agree that the etiology of these health conditions is multicausal and includes a long row of potential risk factors and mediating factors, including those suggested by Dr. Finsterer.

Research questions about causal associations related to the pandemic are highly interesting, and something we hope to contribute to in future articles based on our prospective data where we have followed the therapists over time in the CAMP cohort study. The therapists were included after the outbreak of the pandemic, so unfortunately, we will not be able to shed light on the question “whether psychological stress and musculoskeletal pain have really increased during the pandemic” based on our data.

On behalf of the authors,

Sincerely,

Nathan Weiss.

## Data Availability

Not applicable.

## Abbreviations

COVID-19: Corona Virus Disease 2019
CAMP: Corona and manual professions
